# Multi-Camera Multi-Person Tracking and Re-Identification in an Operating Room

**DOI:** 10.3390/jimaging8080219

**Published:** 2022-08-17

**Authors:** Haowen Hu, Ryo Hachiuma, Hideo Saito, Yoshifumi Takatsume, Hiroki Kajita

**Affiliations:** 1 Graduate School of Science and Technology, Keio University, Tokyo 223-8522, Japan; 2 Department of Anatomy, Keio University School of Medicine, Tokyo 160-8582, Japan; 3 Department of Plastic and Reconstructive Surgery, Keio University School of Medicine, Tokyo 160-8582, Japan

**Keywords:** multi-camera multi-person, pedestrian tracking, human re-identification, operating room, trajectory

## Abstract

Multi-camera multi-person (MCMP) tracking and re-identification (ReID) are essential tasks in safety, pedestrian analysis, and so on; however, most research focuses on outdoor scenarios because they are much more complicated to deal with occlusions and misidentification in a crowded room with obstacles. Moreover, it is challenging to complete the two tasks in one framework. We present a trajectory-based method, integrating tracking and ReID tasks. First, the poses of all surgical members captured by each camera are detected frame-by-frame; then, the detected poses are exploited to track the trajectories of all members for each camera; finally, these trajectories of different cameras are clustered to re-identify the members in the operating room across all cameras. Compared to other MCMP tracking and ReID methods, the proposed one mainly exploits trajectories, taking texture features that are less distinguishable in the operating room scenario as auxiliary cues. We also integrate temporal information during ReID, which is more reliable than the state-of-the-art framework where ReID is conducted frame-by-frame. In addition, our framework requires no training before deployment in new scenarios. We also created an annotated MCMP dataset with actual operating room videos. Our experiments prove the effectiveness of the proposed trajectory-based ReID algorithm. The proposed framework achieves 85.44% accuracy in the ReID task, outperforming the state-of-the-art framework in our operating room dataset.

## 1. Introduction

For purposes of education, research communication, analysis of surgical progress, and so on, it is necessary to record surgical procedures. Most surgical research based on computer vision exploits videos of surgery areas, such as laparoscopic videos [1]. In these videos, only operative incisions and some surgical tools are captured. There are other kinds of surgical videos where the whole operating table can be seen in order to capture the hands of surgical members and more surgical tools, including those that are not being used [2]; however, if we mean to understand what is going on in the operating room, it is not enough only to take videos of the operating table, we also need to know the status of staff inside the operating room; therefore, the entire operating room should be filmed.

**Figure 1:**
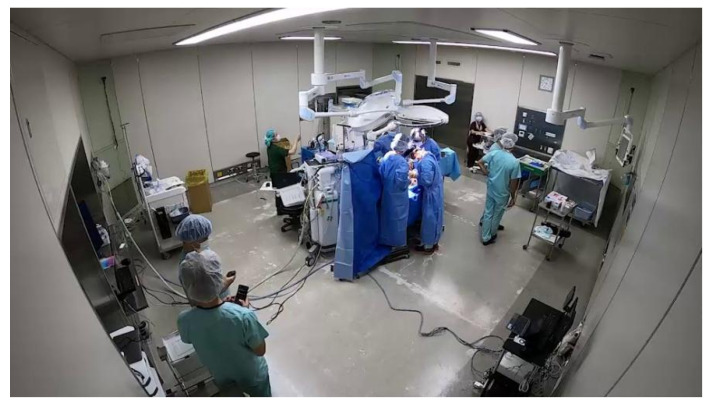
Operating room.

To estimate the locations and movements of staff, we need to track everyone in the room and detect their poses [3,4,5,6]. Hassaballah et al. [7] proposed a robust vehicle detection and tracking approach using a multi-scale deep convolution neural network. Zhou et al. [8] proposed a deep neural network model called A-YONet by combining the advantages of YOLO and MTCNN for end-edge-cloud video surveillance. Chen et al. [9] proposed a pedestrian tracking framework based on faster R-CNN and a full convolution network; however, compared with the usual scenarios, such as street, park, and court, the operating room is quite narrow and full of large instruments (as shown in Figure 1), which means there are lots of blind spots when shooting with only one camera. As a result, we need to record with multiple synchronous cameras; this creates a new problem of how to summarize the content captured by different cameras, which requires inter-camera re-identification (ReID) [10,11,12,13].

In existing methods, some researchers exploit mobile phones [14] and wearable devices [15] to track the movement of pedestrians. Nowadays, the usual way is to extract the image features of pedestrians with convolutional neural networks (CNNs) [16,17,18]. Monocular pedestrian tracking for each camera is conducted first, and then image features of detected bounding boxes in consecutive frames are extracted; finally, these features are compared with those from other cameras to conduct the pedestrian matching, which is called pedestrian ReID.

However, there are some unique characteristics in the scenario of the operating room. There are only a few kinds of surgical uniforms, so several staff members may wear the same kind of uniform with masks at the same time. Moreover, they usually stand very close to each other, which means it is difficult to tell by context. Because of the above points, features extracted by convolutional neural networks are not enough to distinguish among staff members in the same clothes; therefore, we can hardly conduct tracking and ReID with image features directly.

In this paper, we aim to establish a method to obtain reliable monocular staff tracking and inter-camera staff ReID results inside the operating room. Compared to extracting image features with neural networks directly, we mainly judge by the movement trajectories, using texture features as the auxiliary cues. Because the movement of staff members follows certain rules, their trajectories are more reliable than indistinguishable texture features. Even in the cases that someone’s trajectory is “broken” for one camera, which may be caused by occlusions or human detection failures, it is possible to connect their broken trajectories according to more complete trajectories of other cameras during the step of inter-camera ReID. Compared with forcibly connecting two trajectories that might belong to the same person before and after occlusion or other kinds of failure, just in pursuit of better monocular tracking performance, the proposed method can achieve better and more reliable overall performance.

The proposed method can be divided into three steps: first, we detect the poses of staff in every frame captured by every camera with a trained pose-estimation neural network; then, staff members with high pose-confidence scores are screened out, and everyone’s location in the operating room is estimated and tracked according to the image coordinates of their feet and the pre-estimated homography matrix from the image plane onto the world ground plane; finally, we designed a clustering algorithm, re-identifying the detected staff members’ trajectories of all cameras, and then the complete trajectories of all staff members in the operating room can be obtained. In the proposed framework, all involved models do not require training with target scenario data, which means tedious and time-consuming annotation can be omitted, and it is quite convenient for deployment.

The key contributions of this paper are summarized as: (a) we establish a framework integrating monocular tracking and inter-camera ReID; (b) we convert the image coordinates to ground coordinates so that the tracking and ReID tasks can be conducted with actual world distances; (c) we conduct the tracking and ReID tasks with trajectories, taking less distinguishable texture features as the auxiliary cues; (d) we propose a clustering-based ReID method, re-identifying surgical members spatially and temporally; (e) the proposed inter-camera ReID method can not only re-identify surgical members across different cameras, but also conquer the problems such as occlusion during tracking; (f) the proposed framework takes advantage of the high performance of deep learning and machine learning methods in computer vision without the need for training.

The video recording is approved by Keio University School of Medicine Ethics Committee and written informed consent was obtained from the patient; the annotation was conducted by ourselves. We built an operating room dataset for surgical staff tracking and ReID and validated the proposed method with this dataset. The experiments show that the proposed method can deal with occlusions that occur when a staff member is walking and conduct accurate inter-camera ReID when several staff members are wearing the same kind of uniform in the operating room at the same time.

The rest of this paper is structured as follows: Section 2 reviews the related work; Section 3 details the proposed approach; Section 4 discusses experimental results and limitations; Section 5 concludes this work and discusses our future plans.

## 2. Related Work

The pedestrian tracking problem has been a popular research field since it was proposed [19,20]. The ideal case is that everyone is tracked consistently in one video; however, it is not possible in real scenarios because of occlusions, the same person’s leaving and re-entering the view of the camera, and so on. These challenges greatly increase the difficulty of monocular tracking. Until now, the best solution to these challenges is setting up multiple synchronous cameras to make sure the movements of pedestrians are not being missed [21,22]; however, it will also introduce the ReID problem due to the existence of multiple cameras. To settle this problem, researchers have proposed many methods.

Guo et al. [16] proposed a time-based motion model studying the precise time intervals among sub-sampled frames, and an improved multi-person matching cascade scheme to deal with the errors caused by the same person’s leaving and re-entering a camera’s view; however, their method requires a time-consuming training process, and there are high requirements for computing devices even in the inference of models.

Wang et al. [23] presented a network that combines temporal and appearance information as a unified framework. They defined a graph model to associate detection results frame by frame with the help of appearance similarity and spatial consistency, and the similarity is measured by the designed multi-scale neural network. To combine the results corresponding to different cameras, a clustering method is used for ReID. Even though their framework performs well in the scenarios of campus and street, the fast camera motion is still a challenge in 2D tracking.

Nguyen et al. [24] proposed a multi-camera multiple object tracking approach based on a spatial-temporal lifted multi-cut formulation. They refine single-camera tracklets with a novel pre-clustering obtained from 3D geometry projections, then match them to multi-camera trajectories by solving a global lifted multicut formulation that incorporates short and long-range temporal interactions on tracklets located in the same camera as well as inter-camera ones. This framework works well in the square scenario—it is still a challenge when there are noises, occlusions, and some other kinds of interference.

In addition to coming up with better methods, it is also important to build datasets. Kohl et al. [25] created a simulated multi-target multi-camera tracking dataset with a popular game GTA V. Their dataset contains over 2800 person identities, 6 cameras, and a video length of over 100 min per camera. Compared with datasets based on real videos, the simulated dataset is large in terms of the number of identities and video length, and there is no risk of privacy disclosure.

The work most relevant to ours is presented by Lima et al. [26]. They estimated pedestrian location on the ground plane based on human body poses and person’s bounding boxes from an off-the-shelf monocular detector, and then project these locations onto the world ground plane and fuse them with a new formulation of a point-clique cover problem. Their framework has good performance in the scenario of the square; however, in our case, the surgical members in the same clothes stand very closed to each other in a narrow and crowded operating room; therefore, in the proposed framework, the surgical members are detected first; then we conduct monocular tracking for each camera and obtain several broken trajectories; finally, we propose a trajectory-based clustering method to combine the trajectories from different cameras and generate complete trajectories for every surgical member. These modifications help address the problems caused by indistinguishable texture features and integrate spatial and temporal information so that the proposed workflow is more appropriate for the operating room scenario.

## 3. Method Framework

**Figure 2:**
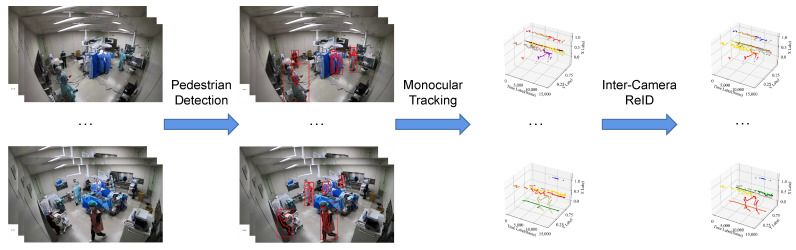
Method flow of the proposed framework.

Because of the particularities of the operating room scenario, we can hardly address the tracking and ReID problems with an end-to-end model; therefore, according to its characteristics, we establish a framework with three models to complete the whole task step-by-step. The method flow is shown in Figure 2. Estimated poses and bounding boxes are utilized to generate several segments of trajectories for all cameras separately and then the obtained broken trajectories are connected and re-identified by combining the results of all cameras.

### 3.1. Pose Estimation and Bounding Box Detection

This is the first step to estimating poses and predicting bounding boxes in the proposed framework. If the detection performance of surgical members is not good enough, it will be difficult for us to conduct the subsequent procedures and obtain reliable results of tracking and ReID; therefore, accurate detection of surgical members who appear, move, and disappear in the videos is vitally important.

Research on human pose estimation has been popular in the field of computer vision for several years [27,28,29]. In this paper, we utilize a mature and popular pose estimation neural network named AlphaPose [27]. For each detected person, AlphaPose gives not only a complete set of pose key points with confidence scores but also a confidence score of the whole pose and their bounding box. With the obtained information, we are able to estimate each surgical member’s location in the operating room and extract their image features in subsequent steps.

### 3.2. Monocular Tracking

After the pose estimation and bounding box prediction, we obtain the necessary prior information to be used in monocular tracking and inter-camera ReID. There are many popular methods for monocular pedestrian tracking that design ingenious structures of neural networks to deal with occlusions or some other challenges in the video, pursuing high monocular tracking performance [3,5].

However, this idea does not make much sense in our case. Due to the particularities mentioned before, methods based on the extracted image features cannot tell surgical members in the same kind of uniforms from each other; therefore, we need to find a more reliable basis to deal with such cases.

Because our final goal is to obtain multi-camera trajectories for all detected surgical members, the monocular trajectories should be less “fallible” rather than more “complete”. For example, when occlusions appear during someone’s movement, it does not matter that their trajectory is broken into several segments, because we can combine these broken segments and obtain a complete trajectory in the step of inter-camera ReID based on trajectories from other cameras. This means that we do not need to deal with occlusions, which might be the biggest challenge in monocular pedestrian tracking.

Therefore, we designed a concise but reliable method inspired by Trackpy [30], utilizing the location of each person in the step of monocular tracking. First, available poses are screened out from all detected ones, deleting those whose pose-confidence score is less than $T_{11}$ or both ankle-confidence scores are less than $T_{12}$.

Then, each screened surgical member’s location is estimated and mapped onto the world ground plane, we estimate one’s location in the image coordinate system, $C_{p}$ = ($x_{p},y_{p}$), with their two ankle coordinates in the same frame, which means we exploit the mean coordinate of one’s two ankles to represent their location:

(1) $$C_{p}=mean\left(C_{la},C_{ra}\right),$$

where $C_{la}$ and $C_{ra}$ represent the coordinates of the left and right ankles, respectively, and we then map $C_{p}$ onto the world ground plane with a pre-estimated homography matrix, *H*, of this camera:

(2) $$s{\left(X_{p},Y_{p},1\right)}^{T}=H{\left(x_{p},y_{p},1\right)}^{T},$$

where ($X_{p},Y_{p}$) represents the coordinate of this person in the ground-coordinate system.

Finally, we track each screened surgical member frame-by-frame, if the distance of their locations in the present frame and next frame is less than a preset threshold $T_{13}$ (the maximum distance a person can run in the ground-coordinate system), they are tracked in next frame. Further, to deal with the cases that someone’s pose may be missing or filtered out in some frames, we set another parameter $T_{14}$ called “memory frame number”, which means we allow for the possibility that a person might be missed for a few frames and then seen again, we keep the track of disappeared surgical members and maintain their IDs for up to some number of frames after their last appearance. In this way, we obtain the initial trajectories of all surgical members for each camera.

### 3.3. Inter-Camera ReID

Because of the occlusions and some other interference, obtained trajectories will be broken into several segments for each surgical member as discussed above. In monocular tracking, this problem is quite hard to settle. Fortunately, it is convenient to place multiple cameras in the operating room scenario, which means we can overcome these challenges with more complete trajectories of other cameras. We introduce the idea of density-based spatial clustering of applications with noise (DBSCAN) [31] to summarize trajectories of different cameras, assigning the same ID to those belonging to the same person.

The idea of the original DBSCAN is quite concise: it starts with an arbitrary core point that has not been visited; this point’s ϵ-neighborhood is retrieved, and if it contains sufficiently many points, a cluster is started; otherwise, the point is labeled as noise. Inspired by this idea, we designed our clustering algorithm: in the original DBSCAN, the core object is defined as a point of high density in its neighborhood, and we define a trajectory whose temporal length (unit: frame) is greater than $T_{21}$ as a core object in our case, based on the completeness of trajectory; for a core trajectory *p*, if the mean distance between *p* and another trajectory *q* is less than $T_{22}$ and the mean bounding box similarity (we use histogram correlation coefficient in this paper) between *p* and *q* is greater than $T_{23}$, *q* is considered an object in the neighborhood of *p*. As for the trajectories labeled as “noise” after clustering, we seek the most possible cluster for each “noise” trajectory based on mean distance and mean bounding box similarity. Further, to avoid logical errors, trajectories that correspond to the same pedestrian and come from the same camera should not overlap in the time domain, since a pedestrian can only appear once in one camera frame. Finally, we obtain all detected surgical members and corresponding trajectories with IDs for each camera.

## 4. Experiments

### 4.1. Dataset

All videos used to build our dataset are actual operating room videos provided by the School of Medicine, Keio University. We have annotated 960 frames in total, they are uniformly sampled from 4 4-minute videos captured by 4 synchronous cameras, respectively. Because the frame rate of provided videos is 60 FPS, dense annotation is not necessary in the case of tracking and it is also extremely time-consuming, we annotate 1 frame for each second. We use this dataset to evaluate the proposed tracking and ReID algorithms.

### 4.2. Implementation of Models

We directly utilize a mature and popular neural network, AlphaPose, to generate poses and bounding boxes with confidence scores for all surgical members of all cameras, frame-by-frame. Yolov3 [32] is used as the pedestrian detector in AlphaPose. In our experiments, we use the weights provided by the author, which are trained on COCO 2017. Because AlphaPose has been proved a reliable pose estimator, we do not conduct quantitative analysis for pose estimation in this paper. According to our observation, the performance is acceptable. Even in the cases of mistakes, most of them can be filtered out by setting confidence thresholds.

In our concise monocular tracking algorithm, surgical members are tracked by their locations mapped from image coordinates to ground coordinates. In our implementation, the four parameters mentioned above, $T_{11}$, $T_{12}$, $T_{13}$, and $T_{14}$, are set as 2, 0.6, 40 cm, and 60 individually. The values of $T_{11}$ and $T_{12}$ are chosen by experience, to filter out mistakes from detected poses. As for $T_{13}$ and $T_{14}$, 40 cm is the length of an adult’s step, and it takes about 1 s (60 frames) to walk a step. In our dataset, the frame number of each video is 14,400. To ensure the completeness of core trajectories, we set $T_{21}$ as 7200, half of the video frame length. Because we need to map the image coordinates of surgical members’ feet of all cameras to the same ground-coordinate system, it is obvious that there will be mistakes during mapping; therefore, we had to take it into account when we were designing the clustering algorithm. According to experiments, it is proper to set $T_{22}$ as 120 cm and $T_{23}$ as 0.6.

### 4.3. Tracking and ReID Performance Evaluation

**Figure 3:**
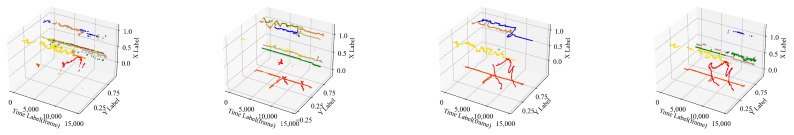
The 3D trajectories of different cameras (separately). The colors in each sub-figure differ among IDs.

With screened poses and bounding boxes, we can conduct monocular tracking for all cameras. As shown in Figure 3, it can be observed that the broken trajectories of some cameras have more complete ones than other cameras. This fact means it is possible to connect the broken trajectories in the following step of inter-camera ReID.

Moreover, we also calculate Multiple Object Tracking Accuracy (MOTA) to measure the overall accuracy of both the tracking and detection:

(3) $$MOTA=1-\frac{FN+FP+IDSW}{GT},$$

where FN and FP represent false negative and false positive samples, IDSW represents ID switches [33], and Identification $F_{1-\mathrm{Score}}$ ($IDF_{1}$) to evaluate the performance of avoiding ID-switches during tracking for each camera:

(4) $$IDF_{1}=\frac{2IDTP}{2IDTP+IDFP+IDFN},$$

compared with MOTA, $IDF_{1}$ takes more ID information into account [34]. As shown in Table 1, it is obvious that the metric values of some cameras are higher than others, which proves our conjecture that the completeness degree of the same person’s trajectories greatly differs among cameras.

**Figure 4:**
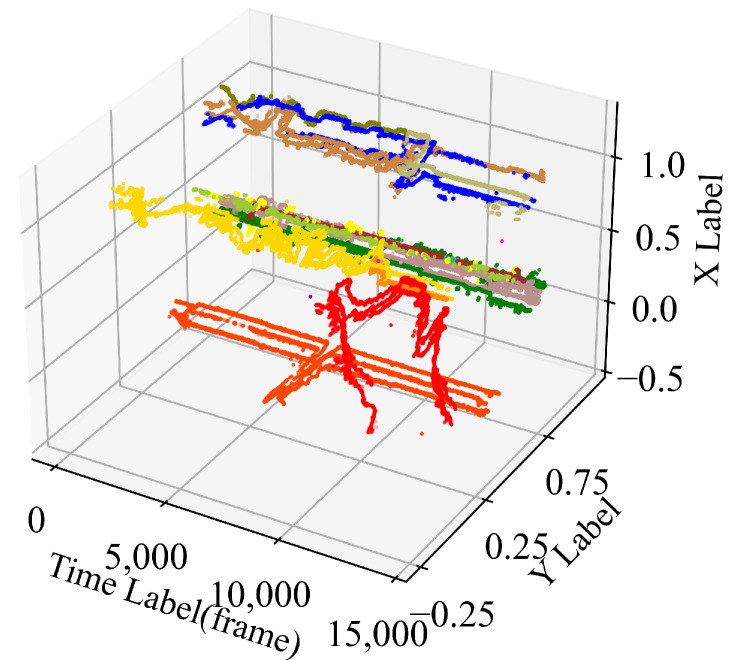
The 3D trajectories of different cameras. The colors differ among IDs.

After monocular tracking, the final step is to combine the results of different cameras and generate much more complete trajectories for all detected surgical members. In Figure 4, we show the 3D trajectories of different cameras after clustering. It can be observed that most trajectories from the same person are assigned the same color (ID), which shows the effectiveness of the proposed framework in MCMP ReID in the operating room.

### 4.4. Discussion and Comparison of ReID

In this part, we discuss the selection of key parameters in the proposed ReID algorithm first, and then compare the proposed framework with a state-of-the-art method [26]. We all utilize AlphaPose as detector and clustering-based algorithms during inter-camera ReID. As introduced before, we need not only to assign a correct ID for each surgical member of all cameras but also to connect broken trajectories during inter-camera ReID; therefore, we calculate Normalized Mutual Information (*NMI*) to evaluate the consistency degree of IDs among cameras:

(5) $$NMI=\frac{2I(Label;Prediction)}{H(Label)+H(Prediction)},$$

where Label and Prediction represent the 1D vectors of labels and predictions—they are generated by connecting the annotated/predicted ID of each person in each frame per camera with making sure both vectors are in the same order; I(Label;Prediction) represents mutual information of the two discrete vectors, which is calculated according to the properties of Kullback–Leibler divergence [35]; H(Label) and H(Prediction) represent the entropy of labels and predictions, individually.

We first compare the results of different sets of key parameters, $T_{22}$ (distance parameter) and $T_{23}$ (similarity parameter), and find out the best set for our ReID method. As shown in Table 2, we find that in the cases that the value of $T_{23}$ is too big (0.8, 0.9), corresponding NMIs are all lower than 78% and quite close, which proves our conjecture that the texture features of surgical members are not distinguishable enough. Further, when the value of $T_{22}$ is too small (40 cm) or too big (200 cm), the ReID accuracy is not satisfying, which is apparent that the improper selection of distance parameter degrades the performance of distance-based clustering. Among all sets above, our method achieves the best performance when $T_{22}$ is 120 cm and $T_{23}$ is 0.6.

**Figure 5:**
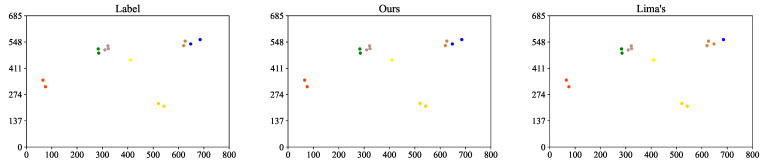
The 2D Comparison of the label, our and Lima’s methods (unit: cm). The colors differ among IDs.

As shown in Table 3, the ReID performance of the proposed framework is better than the state-of-the-art framework. The biggest difference is that ReID is conducted frame-by-frame in Lima’s method, which means the objects to be fused are single points, the temporal information is not taken advantage of in their case; in contrast, we conduct monocular tracking first after human detection, and then fuse obtained trajectories. In other words, we not only utilize spatial information and texture features but also integrate temporal information which we believe is quite helpful for the ReID task. As the example shows in Figure 5, the ReID result of our method is different from Lima’s, and ours is the same as the label. While in Lima’s result, even though the three brown points seem to belong to the same person, they actually do not, and such mistakes are quite hard to avoid without temporal information. It proves the superiority of using trajectory rather than a single point as the unit of the ReID task.

### 4.5. Limitation

**Figure 6:**
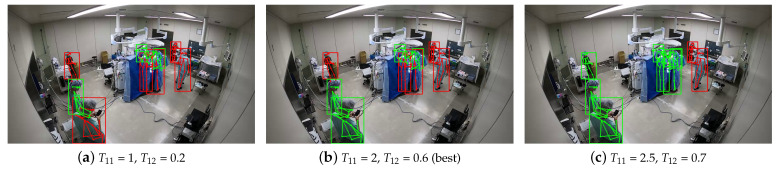
Estimated poses of corresponding bounding boxes. The green poses and bounding boxes represent those being filtered out, and the red ones represent those being kept.

Because the proposed framework starts with pedestrian detection, the accuracy of detection will of course affect the overall performance of the proposed framework. Though we designed a trajectory-based workflow to deal with occlusions and other challenges, the mistakes that happen in pedestrian detection caused by severe occlusions (especially when the surgical members are in the same kind of uniforms) cannot be ignored. In Figure 6b, it is obvious that the estimated poses and corresponding bounding boxes in green are wrong, and they are filtered out with the best confidence thresholds so that these mistakes will not mislead subsequent tracking and ReID. In Figure 6a, a “bad” detection in the bottom left corner is kept with low thresholds; in Figure 6c, many “good” detections are filtered out when the thresholds are set too high; therefore, it is important to set proper confidence thresholds for the detected poses and bounding boxes.

**Figure 7:**
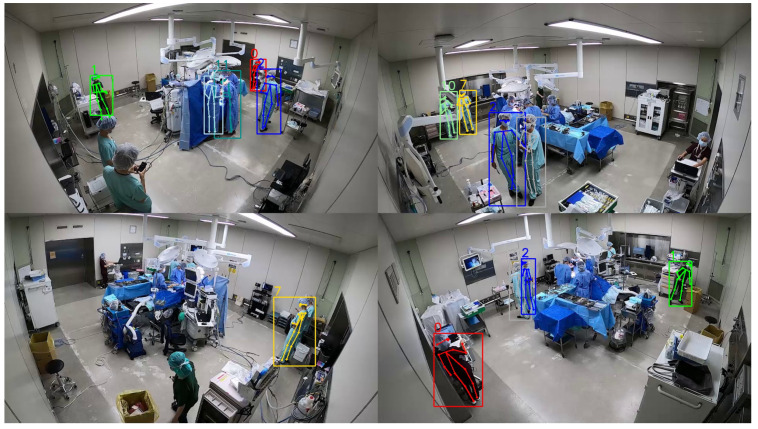
Pictures captured by different cameras with human IDs.

However, even though we have set the confidence thresholds properly, sometimes they still result in many available detected surgical members being filtered out along with the mistakes. As shown in Figure 7, the IDs of some surgical members are not given in some pictures. This problem is mainly caused by the inaccuracy of pedestrian detection and the difference between surgical-member images and common pedestrian images. Because it is extremely tedious to annotate human poses frame-by-frame in operating room video, we directly utilize a pre-trained pose detection neural network with the weights provided by its authors, without being trained in our own dataset. Since we have to use a rough way to screen all detected persons, some available ones have been filtered out even before the tracking and ReID stages, and this problem is hard to solve within the present framework.

## 5. Conclusions and Future Work

In this paper, we establish a framework to track and re-identify each surgical member captured by multiple cameras in a narrow and crowded operating room full of obstacles. A mature and popular neural network is first utilized to detect necessary poses and bounding boxes. Against the particularities of the operating room scenario, we propose a location-based monocular tracking method to obtain several segments of trajectories for each person of each camera and a trajectory-based clustering algorithm for the ReID task. All models in the proposed MCMP tracking and ReID framework do not require training before deployment, freeing users from the tedious and time-consuming annotation task. Compared with the state-of-the-art method, we take advantage of temporal information in our framework, which leads to more reliable ReID results. The proposed framework achieves a higher value of 85.44% than that of the state-of-the-art method (77.56%) on NMI, proving the effectiveness of the proposed trajectory-based ReID ideas and the superiority of the proposed framework compared with the state-of-the-art method.

In future work, we plan to design a trainable automatic selection algorithm for all involved super-parameters to learn the potential relations among parameters, refining the overall performance of monocular tracking and inter-camera ReID. The algorithm should be adapted to the scenario of the operating room. We believe it will help make the proposed framework more broadly applicable to different scenarios.

## Figures and Tables

**Table 1:** MOTA and $IDF_{1}$ of different cameras.

Camera	1	2	3	4
MOTA (%)	74.64	87.02	92.43	87.34
$IDF_{1}$ (%)	79.19	80.44	100.00	79.22

**Table 2:** NMI (%) corresponding to different sets of key parameters.

	$T_{23}$	0.5	0.6	0.7	0.8	0.9
$T_{22}$ (cm)						
40		78.71	81.53	77.85	77.85	77.85
80		79.16	82.54	79.15	77.12	76.51
120		79.16	**85.44**	81.61	77.68	77.85
160		80.25	82.62	78.91	76.14	77.93
200		75.69	78.93	75.86	74.70	74.76

**Table 3:** NMI of the proposed ReID method and state-of-the-art method.

Method	Ours	Lima’s
NMI (%)	85.44	77.56

## Data Availability

The data reported in this study are not available because they are private data from Keio University.

## Abbreviations

The following abbreviations are used in this manuscript:

MCMP	Multi-camera multi-person
ReID	Re-identification
CNNs	Convolutional neural networks
DBSCAN	Density-based spatial clustering of applications with noise
MOTA	Multiple object tracking accuracy
IDF${}_{1}$	Identification F${}_{1-\mathrm{score}}$
NMI	Normalized mutual information
